# Tracking Toxins: A Pilot Investigation of Cyanotoxins in North-Central Tennessee’s Surface Waters and Wells

**DOI:** 10.3390/toxins18060239

**Published:** 2026-05-22

**Authors:** Kristi L. Hill, Andrea C. Jaegge, Devin M. Moore, Thomas D. Byl

**Affiliations:** 1 Lower Mississippi Gulf Water Science Center, U.S. Geological Survey, 640 Grassmere Park STE 100, Nashville, TN 37211, USA; tdbyl@usgs.gov; 2 Division of Water Resources, Tennessee Department of Environment and Conservation, 500 James Robertson Parkway, Nashville, TN 37243, USA; andrea.jaegge@tn.gov; 3 Department of Environmental Sciences, College of Agriculture, Tennessee State University, Main Campus 3500 John A Merritt Blvd, Nashville, TN 37209, USA

**Keywords:** cyanobacteria, cyanotoxins, CyanoHABs, microcystin, anatoxin, cylindrospermopsin, saxitoxin, water quality, freshwater

## Abstract

Cyanobacterial toxins (cyanotoxins) threaten aquatic ecosystems and human health, yet the factors influencing their production and distribution in freshwater remain unclear. In north-central Tennessee, nutrient-rich runoff from agricultural and urban areas, combined with a karst landscape that supports drinking and recreational water use, heightens the need to understand cyanotoxin behavior. To examine cyanotoxin patterns, the U.S. Geological Survey and the Tennessee Department of Environment and Conservation monitored 18 sites, including two wells under the influence of surface water, every two weeks from September 2022 to November 2024. At least one cyanotoxin was detected at all sites, with the highest concentrations in deep reservoirs and lower levels in shallow systems. Most detections occurred during summer and fall, aligning with high temperatures and rapid-onset drought. Statistical analysis indicated that increased specific conductivity and pH raised the likelihood of detecting total microcystin, likely resulting from drought conditions and nutrient-laden runoff. Additionally, dissolved microcystin showed an inverse relationship with Cumberland River water levels, and principal component analysis showed that Secchi depth, chlorophyll a, pH, temperature, and conductivity explained most water quality variability. These results help increase understanding of cyanotoxin distribution and associated water quality conditions during detections to guide future freshwater cyanotoxin monitoring studies.

## 1. Introduction

Cyanobacterial harmful algal blooms (CyanoHABs) pose a significant threat to drinking water supplies [1,2], recreational activities [3], and aquatic ecosystem health [4] worldwide. In addition to the potential non-toxic impacts of CyanoHABs, which include oxygen depletion [5], altered food web dynamics [6,7], and esthetic concerns [8,9,10], many genera of cyanobacteria can produce toxic secondary metabolites (cyanotoxins), which include some of nature’s most toxic compounds [11,12], such as *Dolichospermum* sp. [13], *Anabaena*, *Raphidiopsis* (formerly *Cylindrospermopsis*) [14], and *Microcystis* [15]. Depending on the taxonomic composition of a given freshwater CyanoHAB, neurotoxins (i.e., saxitoxin and anatoxin) and/or hepatotoxins (i.e., microcystin [MC], nodularin, and cylindrospermopsin) may be produced and subsequently released into the environment [11]. While increased temperatures [15,16,17,18], longer residence times [15,16,17], higher pH values (>7) [14,18], and eutrophication [18,19,20] are all thought to favor potential CyanoHAB formation and subsequent toxin release, these events remain unpredictable and episodic [20,21,22,23,24,25,26]. Limited monitoring studies have revealed that drivers vary in time and location based on regional conditions, such as weather, and variability among waterbodies (i.e., depth, discharge, nutrient inputs) [2,4,13,22,27]. Because the scale, frequency, and duration of reported CyanoHAB incidences are increasing [28,29] and are posing an increasing threat to inland freshwater systems [12], there is an emerging need to increase the present understanding of the drivers of toxin production, occurrence, and distribution in freshwater systems. This information can be used to help assess the risks presented by CyanoHABs in this region and refine routine monitoring and HAB response programs.

**Figure 1:**
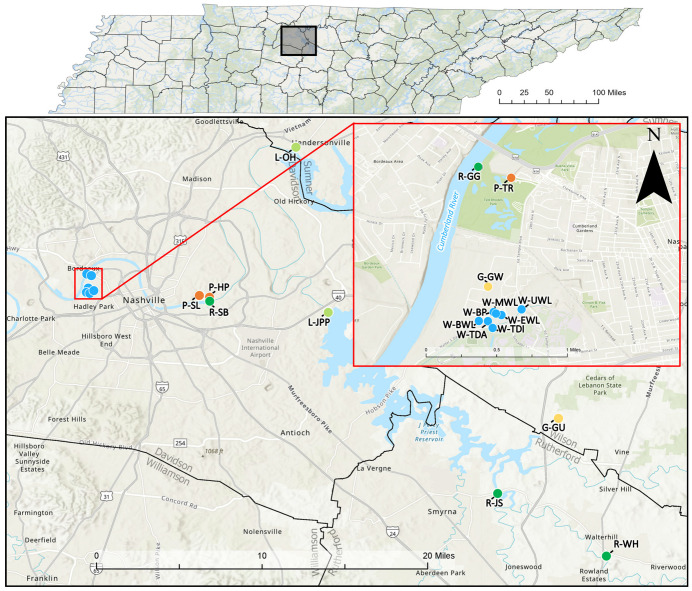
Map showing cyanotoxin monitoring site locations around Nashville, TN. The study area is defined approximately by the shaded black box on the above county map of the state of Tennessee. Sites are color-coded by waterbody type: lake = light green, pond = orange, river = green, groundwater = yellow, wetland = blue. Base map sources: Esri, DeLorme, HERE, TomTom, Intermap, increment P Corp., GEBCO, USGS, FAO, NPS, NRCAN, GeoBase, IGN, Kadaster NL, Ordnance Survey, Esri Japan, METI, Esri China (Hong Kong), swisstopo, MapmyIndia, and the GIS User Community.

In north-central Tennessee, CyanoHABs are historically understudied but remain a pressing concern due to the region’s diverse waterbodies—including rivers, ponds, wetlands, and lakes—which are essential for ecological and economic health [30,31,32]. The Cumberland River watershed serves as the major source of drinking water for communities throughout north-central Tennessee, and numerous tributaries and impoundments on the river are regularly used for recreation, industry, and agriculture [30,33]. Nashville is the most densely populated area in the Cumberland River watershed, and public-supply water demand on the watershed is projected to increase between 60% and 130% by 2040 [33]. Additionally, several species that are federally listed as threatened or endangered, including the endangered Nashville crayfish (*Orconectes shoupi*) [34] and the proposed endangered Cumberland moccasinshell (*Medionidus conradicus*) [35], inhabit this area of Tennessee. Previous freshwater cyanotoxin monitoring studies have found inverse correlations between CyanoHAB occurrences and aquatic species abundance [36,37]. Specifically, populations of crayfish and freshwater mussels have been negatively affected during cyanotoxin occurrences in freshwater systems [38,39]. Furthermore, the karst landscape of north-central Tennessee is characterized by sinkholes, caves, and springs [40] and raises additional concerns regarding cyanotoxins in groundwater because these features can allow surface water mixing in groundwater [41,42,43]. While CyanoHABs have not been a major concern in the region until the late 1900s [31], the increasing stress and demand for water resources coupled with the ecological vulnerability of the region’s unique biodiversity and landscape underscores the need to monitor a diverse array of aquatic ecosystems to better understand the drivers and risks of CyanoHABs and related toxin production and distribution. To improve the understanding of cyanotoxin distribution, occurrence, and drivers for toxin production in freshwater systems, the U.S. Geological Survey (USGS), in collaboration with the Tennessee Department of Environment and Conservation (TDEC) and Tennessee State University (TSU), conducted a two-year monitoring study (2022–2024) across 18 sites (Figure 1) to characterize the distribution and occurrence of cyanotoxins in a variety of waterbody types in north-central Tennessee.

The following objectives were identified to address the goals of this study: (1) assess the spatial and temporal distribution of cyanotoxins across north-central Tennessee in various waterbody types, and (2) identify physiochemical factors that may influence cyanotoxin presence. Toxins were sampled using both discrete grab samples and time-integrated Solid Phase Adsorption Toxin Tracking (SPATT) samplers [44], which have been shown to be an effective method for quantifying cyanotoxins and monitoring dynamics during and in-between discrete site visits [45,46]. The findings from this study provide preliminary insight into the drivers of cyanotoxin dynamics and help characterize conditions in which CyanoHAB impacts are more likely to develop and persist in north-central Tennessee and other regions with similar humid-subtropical climates and freshwater ecosystems. A better understanding of the distribution and drivers for cyanotoxin production in wetlands, ponds, rivers and reservoirs will allow for more effective mitigation and management of CyanoHABs in the future.

## 2. Results

### 2.1. Spatial Distribution of Microcystin

**Figure 2:**
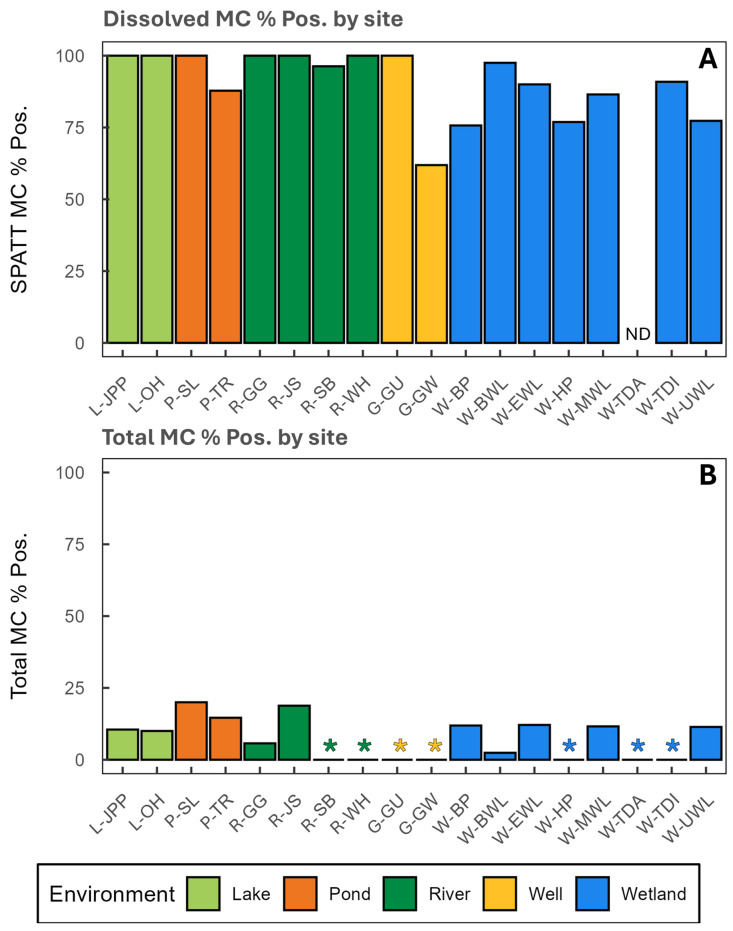
Bar plots showing (**A**) SPATT (Solid Phase Adsorption Toxin Tracking) sampler-derived percentages of samples with detectable (>0.150 µg/L) dissolved MC (% Pos.) and (**B**) discrete water sample-derived percentages of samples with detectable (>0.150 µg/L) total MC (% Pos.) plotted by site. Bar colors represent the associated sampling environment. ND indicates that no data were collected at a site. “*” indicates that all samples were below the MC reporting range, meaning the site had 0% positive detections.

Eighteen monitoring sites were selected with consideration of water utility intakes, popular recreational areas, and location accessibility; the original assigned site names were labeled with a site type prefix indicating the waterbody type they were grouped with. The waterbody type labels and prefixes are as follows: lake (L-), pond (P-), river (R-), groundwater (G-), and wetland (W-). All 17 of the sites monitored using SPATT samplers experienced detectable levels of dissolved MC at some point during the study period. Dissolved MC was less consistent and was detectable in every SPATT sampler deployed at sites L-JPP, L-OH, P-SL, R-GG, R-JS, R-WH, and G-GU (Table 1; Figure 1). The highest concentrations of dissolved MC were measured at sites L-JPP, R-SB, and P-TR (Table 1). The lowest concentrations of dissolved MC were measured at sites G-GW, W-HP, W-TDI, and W-EWL (Table 1). An additional parameter, referred to as percent positive, was calculated by dividing the number of detections by the total number of samples at a given site and expressing the result as a percentage that is representative of the ratio of detectable MC occurrences for each site (Table 1 and Table 2) and each month (Table 3). The lowest percentages of positive dissolved MC detections occurred at sites G-GW, W-BP, W-HP, and W-UWL (Table 1; Figure 2).

Grab samples showed no detectable total MC concentrations for the duration of the study for sites R-SB, R-WH, G-GU, G-GW, W-HP, W-TDA, and W-TDI (Table 1; Figure 2). The highest ratios of detectable total MC occurred at sites P-SL, R-JS, and P-TR (Table 1; Figure 2). The W-BP site and the W-UWL site had the widest range of total measured MC concentrations, showing sample concentrations ranging from non-detection to exceeding the upper reporting limit, though their medians indicate that total MC concentrations typically remained below detectable concentrations (Table 1).

### 2.2. Spatial Distribution of Anatoxin, Cylindrospermopsin, and Saxitoxin

Subsets of SPATT samplers were analyzed for additional cyanotoxins. Anatoxin was detected at 100% of sites tested, cylindrospermopsin was detected at 90% of sites tested, and saxitoxin was detected at approximately 71% of sites tested (Table 2). The highest percentage of saxitoxin detections occurred in wetland sites, followed by ponds, wells, rivers, and lakes, respectively (Table 2). The anatoxin maxima exceeded the reporting limit (25 µg/g) at sites L-JPP, P-TR, R-GG, R-JS, R-SB, R-WH, R-GU, W-BP, W-EWL, and W-UWL; anatoxin medians exceeded 10 µg/g at sites G-GU and L-JPP (Table 2). The cylindrospermopsin maxima exceeded 2.0 µg/g at sites W-BWL, W-EWL, and W-MWL. The measured saxitoxin maxima occurred at the P-TR and L-OH sites; saxitoxin medians were below detection at all sites tested except for the W-HP site (Table 2).

Cylindrospermopsin was found in all the SPATT samplers tested except for the G-GW site, indicating that cylindrospermopsin is a prevalent cyanotoxin in north-central Tennessee surface waters. The neural toxin, anatoxin, was present in all the SPATT samplers tested from the lake and river sites, and most of the SPATT samplers from the pond and wetland sites. Saxitoxin presence was less prevalent and mostly detected inconsistently over time in the shallow wetland sites. This documented widespread occurrence and the significant health risks caused by anatoxin and cylindrospermopsin provide support for the inclusion of anatoxin and cylindrospermopsin into monitoring programs in north-central Tennessee and similar ecoregions.

### 2.3. Temporal Distribution of Microcystin

**Figure 3:**
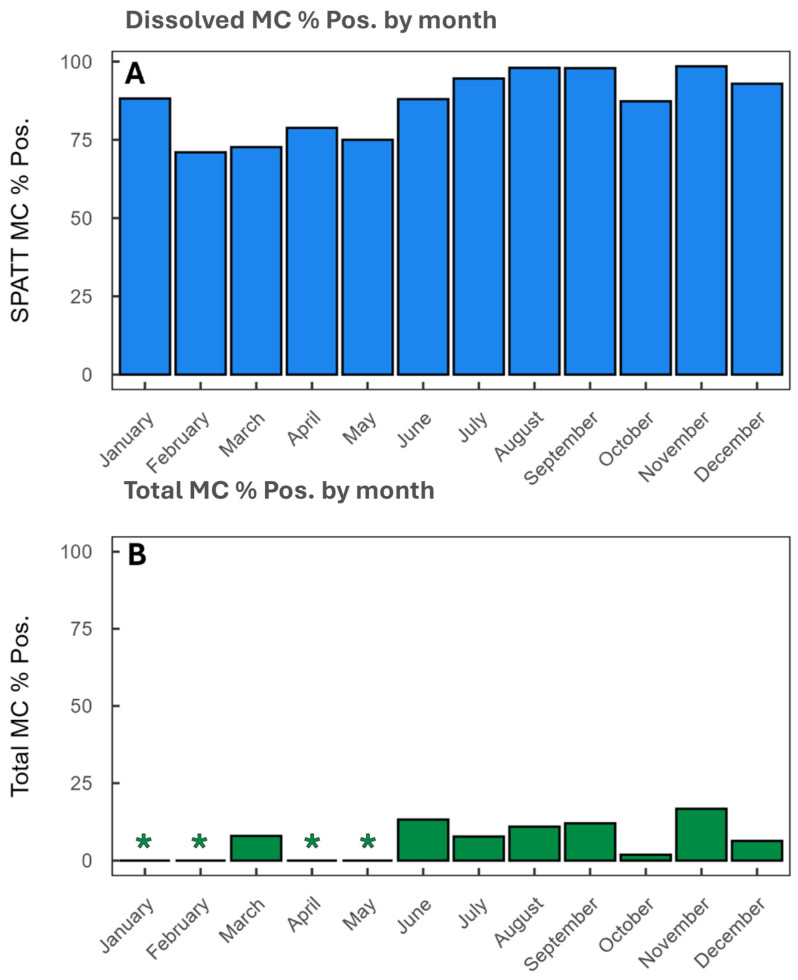
Bar plots showing (**A**) SPATT sampler-derived (blue) percentages of samples with detectable (>0.150 µg/L) MC (% Pos.) and (**B**) mean discrete water sample-derived (green) percentages of samples with detectable (>0.150 µg/L) total MC (% Pos.) across each sampling month. “*” indicates that all samples were below the MC reporting range, meaning the site had 0% positive detections.

**Figure 4:**
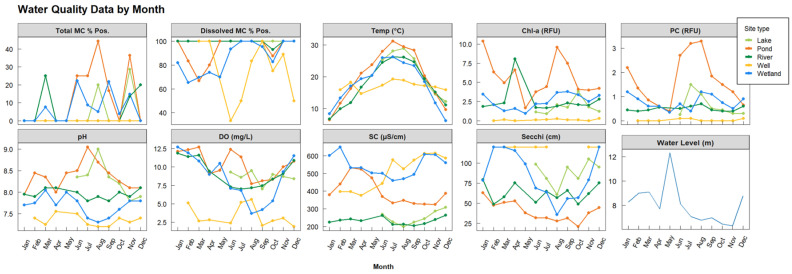
Multi-panel plot of total MC positive detections (% Pos.), dissolved MC positive detections (% Pos.), temperature medians, Chl-a medians, PC medians, pH medians, DO medians, SC medians, Secchi medians, and maximum water level values pooled by month. The associated water quality field data used to generate these plots are publicly available from [47].

All data were pooled by month for the duration of the study to investigate evidence for general temporal trends. Total MC was not detected during January, February, April and May (Table 3; Figure 3). The highest dissolved MC concentrations were measured in September, August, and November, and the percent positive ratios of dissolved MC detections exceeded 90.0% in July, August, September, November, and December (Table 3; Figure 3 and Figure 4). Dissolved MC did not exceed 10 µg/g in January, February, March, or May (Table 3). Detectable concentrations occurred in June and continued through December (Table 3), correlating with warmer temperatures from summer through late fall in north-central Tennessee (Table 3). The highest percentages of positive total MC detections occurred in November, June, and September, with a notable decrease in detections in October (Table 3; Figure 3 and Figure 4). Water level (gage height) changes from the continuous USGS water level monitoring station 03431091 (station name: Cumberland River at Omohundro Water Plant at Nashville, TN [48]) were used to indicate increases and decreases in precipitation. Water levels were highest in May, March, and February and lowest in November, October, and August (Table 3; Figure 4). These findings support an apparent inverse relationship between water levels and MC concentrations.

### 2.4. Water Quality Data

Water quality field data collected during each site visit were used to characterize variability among monitoring sites and statistically analyzed to identify potential relationships associated with elevated cyanotoxin. Sites were classified by depth as either wadable or non-wadable, forming three groups: shallow (ponds, wetlands), deep (lakes and rivers), and wells. The highest water temperatures were measured at shallower surface water sites, primarily wetlands, and the lowest temperatures were measured at the two well sites (Table 4). The highest median dissolved oxygen (DO) concentration was measured at the W-MWL site; the G-GW site had the lowest median DO concentration (Table 4). The DO concentrations at the W-HP site were the most variable as indicated by a high standard error (Table 4). The G-GU site had the largest range in specific conductance (SC), possibly due to surface water mixing with the groundwater during heavy rains in the karst terrain (Table 4). The shallow sites typically had higher measured SC values and SE than the deep sites (Table 4). A positive relationship between chlorophyll a (Chl-a) and phycocyanin (PC) pigments was identified at most sites (Table 4); however, in Kendall’s Tau correlation tests of the pigment concentrations, Chl-a showed a more significant relation to MC detections than PC (tau_Chl-a_ = 0.12, *p*-value_Chl-a_ = 0.001; tau_PC_ = 0.09, *p*-value_PC_ = 0.02). The highest total Chl-a medians were observed at sites P-SL, W-HP, W-BP, P-TR, and W-MWL (Table 4). The highest PC medians were observed at sites W-HP, P-SL, W-BP, P-TR, and L-OH (Table 4). These sites were all the shallowest ponds or wetlands with the slowest discharges relative to the other monitoring sites, making them more vulnerable to sporadic eutrophication. The high SE values for most water quality parameters at the pond and wetland sites also indicate high water quality variability at these site types (Table 4). The two groundwater (well) sites and the W-BWL site had the lowest Chl-a and PC fluorescence medians (Table 4). The most variable Chl-a and PC fluorescence values were measured at the W-HP site, a shallow ephemeral wetland with very low discharge and little mixing (Table 4). The higher variability of water quality in shallow sites versus deep sites was confirmed by applying a general variability test to the three depth groups (shallow, deep, and wells), in which shallow sites showed a significantly larger general variance and top-k value than deep sites (Table 5). For all field data, the shallow sites had higher SE values, indicating higher water quality variability at these site types compared to the deep sites (Table 4).

**Figure 5:**
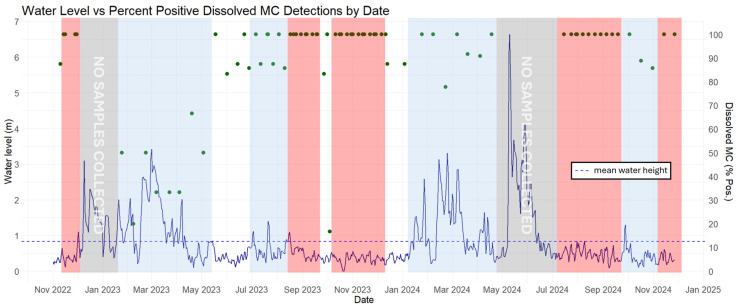
Time series plot of samples with detectable levels of dissolved MC (% Pos.) from SPATTs measured during 2022–2024 and the corresponding water level (gage height) from USGS stream gage 03431091 (Cumberland River at Omohundro Water Plant at Nashville, TN). Water level (gage height) data are publicly available from [48]. Water levels were normalized to the origin (i.e., the lowest water level reported = 0 m) using reported water levels from continuous streamgage data and are represented by a dark blue line. The dashed blue line represents the mean water height for the streamgage for the study period; the green points represent percent positive (% Pos.) dissolved MC detections by date sampled across the study period. Red boxed portions represent periods of below-average water levels and 100% Pos. dissolved MC detections; blue boxed portions represent periods of above-average water levels and decreased dissolved MC detections; gray boxed portions represent periods in which no samples were collected.

Peak temperature was measured in June, with the next highest maximum values occurring in July, August, and September, respectively (Table 6). The lowest maximum temperatures were measured in January, February, November, and December, respectively; January and December also had the lowest median temperatures (Table 6; Figure 4). High median DO concentrations in December, January, February, March, and May were measured during increased seasonal precipitation events (Table 3 and Table 6; Figure 4 and Figure 5), indicated by water level peaks captured at USGS streamgage 03431091 [48]. High DO concentrations are also typical during these cooler months due to the higher solubility of oxygen, which decreased as water temperatures increase in the summer. Median pH values only exceeded 8.0 in March and May; median pH values were lowest in July and August (Table 6; Figure 4). The Secchi depth medians exceeded the reporting limit (120 cm) in March and December (Table 6; Figure 4).

### 2.5. Cyanotoxin Synthetase Genes

**Figure 6:**
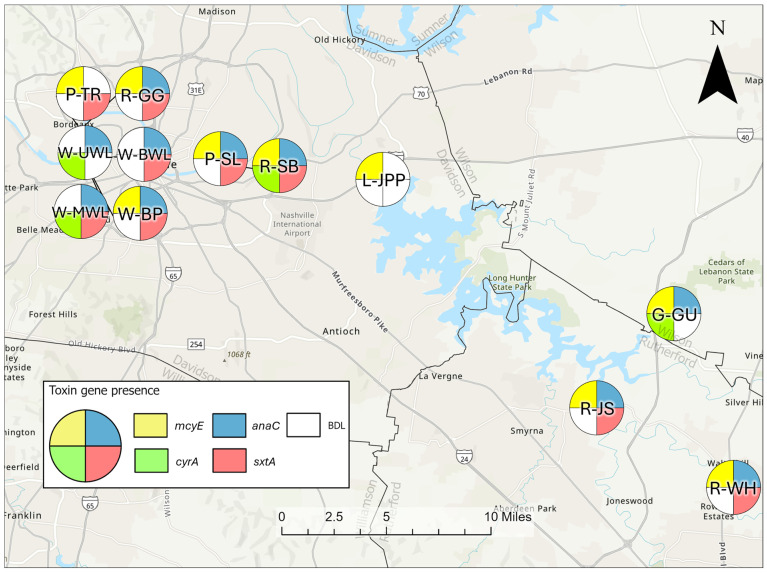
Map showing toxin gene presence from water samples collected in August 2023. Toxin synthetase genes analyzed included *anaC* (blue), *mcyE* (yellow), *cyrA* (green), and *sxtA* (red); white quarters represent the associated synthetase gene as below the quantification limit (<QL; 1100 copies/100 mL for *mcyE*, 60 copies/100 mL for *anaC*, 120 copies/100 mL for *cyrA*, and 300 copies/100 mL for *sxtA*). Letters in circle plots are site identifiers described further in Table 1. Base map sources: Esri, DeLorme, HERE, TomTom, Intermap, increment P Corp., GEBCO, USGS, FAO, NPS, NRCAN, GeoBase, IGN, Kadaster NL, Ordnance Survey, Esri Japan, METI, Esri China (Hong Kong), swisstopo, MapmyIndia, and the GIS User Community.

Twelve sites were sampled for analysis of cyanobacteria 16S ribosomal RNA (rRNA) genes and cyanotoxin synthetase (mcyE, anaC, cyrA, sxtA) gene abundances using quantitative polymerase chain reaction (qPCR). Cyanobacteria 16S genes were present at all sites with abundances ranging from 1.8 × 10^6^ copies per 100 mL to 2.2 × 10^9^ copies per 100 mL (Table 7). The mcyE gene was present at 75% of the sampled sites, anaC at 83.3% of sites, cyrA at 33.3% of sites, and sxtA at 75% of sites (Table 7; Figure 6). All 12 sites contained at least one of the four toxin synthetase genes at the time of sample collection in August 2023 (Table 7; Figure 6). The maximum observed mcyE and cyrA gene abundances were measured at the R-SB site (Table 7). The maximum gene abundance for anaC was measured at the W-UWL site, and the maximum sxtA gene abundance was measured at the R-JS site (Table 7). The maximum abundance of the sxtA gene was 100-fold higher than the other sites analyzed (Table 7). The L-JPP and G-GU sites were relatively low in all gene abundance compared to the other sites (Table 7).

### 2.6. Physicochemical Drivers of Microcystin Production

**Figure 7:**
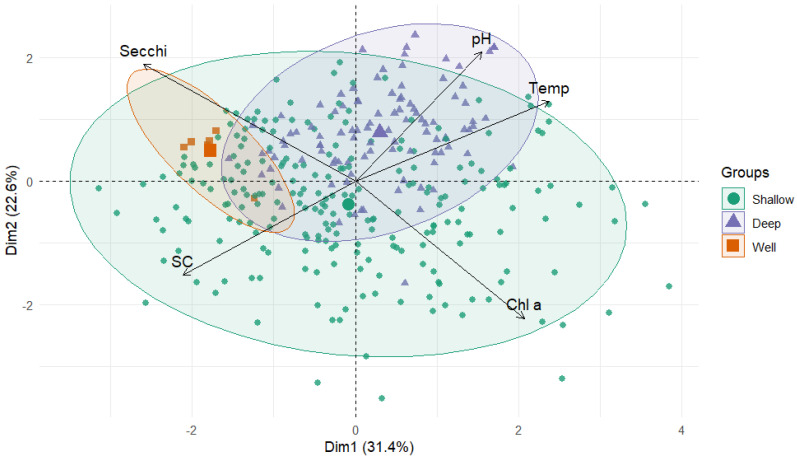
Principal component analysis (PCA) of environmental parameters at sampling sites throughout north-central Tennessee. Color and shape represent the depth grouping of the sampling site and relative size indicates the contribution of an observation. Temp. = temperature, Chl a = Chlorophyll a fluorescence, SC = specific conductance, and Secchi = Secchi depth.

Five notable periods occurred during which water levels remained below average at USGS streamgage 03431091 [48]: November 2022, September 2023, November–December 2023, August–September 2024, and December 2024 (Figure 5). Detectable MC was present in all samples collected during these periods (Figure 5). The highest frequency of samples with non-detectable MC concentrations occurred during periods of peak water height at streamgage 03431091, which occurred February 2023–June 2023 and January 2024–April 2024 (Figure 5). A Kendall rank correlation coefficient was calculated for the streamgage water levels (gage heights) and MC detection variables, confirming a significant moderate inverse correlation between MC presence and water levels (tau = −0.16, *p*-value = 0.04). The second principal component (PC2) explained an additional 22.5% of the variation and highlighted the importance of chlorophyll a (Table 8; Figure 7). Together, PC1 and PC2 account for over 54.1% of the cumulative variation (Table 8; Figure 7). The third principal component (PC3), which includes pH, contributes 18.8% to the variation (Table 8; Figure 7). The fourth principal component (PC4), encompassing temperature and conductivity, accounts for an additional 15.8% of the variation (Table 8; Figure 7).

The first principal component (PC1) identified accounted for 31.6% of the total variation in field water quality parameters and was primarily influenced by Secchi depth (Table 8). The second principal component (PC2) explained an additional 22.5% of the variation and highlighted the importance of chlorophyll a (Table 8; Figure 7). Together, PC1 and PC2 account for over 54.1% of the cumulative variation (Table 8; Figure 7). The third principal component (PC3), which includes pH, contributes 18.8% to the variation (Table 8; Figure 7). The fourth principal component (PC4), encompassing temperature and conductivity, accounts for an additional 15.8% of the variation (Table 8; Figure 7). The logistic regression between the principal components and MC detections showed a relationship with shifts in cyanotoxin occurrence. Logistic regression results were interpreted as odds ratios > 1 (suggesting higher probability of MC presence based on total MC data) and <1 (suggesting reduced probability of MC presence based on total MC data; Table 9). The results indicated that SC (odds ratio = 1.003; *p* = 0.004) and pH (odds ratio = 2.26; *p* = 0.025) were significant predictors of total MC presence (Table 9). For every unit increase in SC and pH, the probability of total MC presence across north-central Tennessee increases by 0.3% and 126%, respectively (Table 9).

## 3. Discussion

### 3.1. Cyanotoxin Dynamics

This study presents a preliminary assessment of cyanotoxin occurrence and distribution using water samples collected across a diverse range of waterbody types in north-central Tennessee over a two-year period. During the study period, all 18 monitoring sites experienced detectable levels of MC, confirming the widespread presence of microcystin in various freshwater ecosystems across the region. Temporal patterns of MC occurrence were generally similar between SPATT samplers and discrete grab samples; however, MC concentrations in SPATT samplers were typically much higher than those in grab samples, which was to be expected as the SPATT method is time-integrated. Discrete grab samples analyzed for total MC typically showed no detectable MC, and the few detectable occurrences rarely exceeded the 0.3 µg/L drinking advisory limit set by the U.S. Environmental Protection Agency (EPA) [49]. In contrast, SPATT samplers had detectable dissolved MC for the entire study period at seven of the 17 sites monitored (L-JPP, L-OH, P-SL, R-GG, R-JS, R-WH, G-GU). The seven sites cover a variety of waterbody types including lakes, ponds, rivers, and wells, indicating a persistent presence of cyanotoxins across diverse aquatic environments in north-central Tennessee. Notably, two of the seven sites (R-WH and G-GU) never had detectable levels of total MC in grab samples despite the recurring detectable dissolved MC in SPATT samplers. This discrepancy implies that different waterbody types support variable frequencies of CyanoHABs and levels of cyanotoxin concentrations [50,51,52,53] that discrete sampling cannot reliably capture. Discrepancies may also be attributed to the location of sample collection and number of samples collected at each site, but due to the exploratory premise of this study to confirm the presence of cyanotoxins, full waterbody characterization by site and consistent sample sizes were not prioritized in the study design. Inadvertently, these identified discrepancies confirm that the frequency, length, and toxicity of CyanoHABs may be influenced by the type of waterbody. Additional research would be needed to characterize the differences across waterbody types, with a primary focus on discharge and depth. These discrepancies also raise concerns about potential chronic exposure and bioaccumulation risks, particularly for waters near public intakes or recreational areas, because microcystins have a half-life of weeks to months depending on environmental conditions [54,55].

The highest concentrations of dissolved MC in SPATT samplers were observed at a lake (L-JPP) and river site (R-SB), two of the deep sample sites. At the L-JPP site, in addition to relatively high dissolved MC, genetic analysis detected a single cyanotoxin gene, (*mcyE*), which is associated with MC production. L-JPP also had the lowest gene detection values for all other toxin genes, compared to the other surface water sites sampled. Additional monitoring with more frequent discrete sampling, cyanotoxin gene analysis, and a benthic sampling component could improve understanding of anatoxin dynamics. Several wetland sites (W-HP, W-BP, W-TDI, W-UWL, W-MWL, and W-EWL) had low median dissolved MC concentrations, likely due to the inherent reduced residence times in these shallower waterbodies. In shallow waterbodies, blooms are more susceptible to flushing and/or co-precipitating with suspended sediments during storm runoff events [50,56,57,58]. In 2023, Cotton and Byl measured detectable MC concentrations in wetland sediments near the W-EWL and W-MWL sites, confirming additional cyanotoxin presence in the wetland benthos [59], suggesting the possibility that toxin concentrations from the water column may be reduced due to the deposition of toxin in underlying sediments, therefore supporting this premise [37,50,51,60,61].

Despite the low dissolved MC found in the SPATT samplers, several wetland sites (W-BP, W-MWL, and W-UWL) occasionally had high total MC, with concentrations exceeding 1 µg/L in grab samples. This contrast highlights the dynamic nature of shallow systems, in which MC production seems to be more sensitive to changing environmental conditions. Evidence of increased algal growth was confirmed with the increase in chlorophyll a and phycocyanin observed in the field data. Shallow systems such as wetlands and ponds are often more productive than other aquatic environments [62], and due to their low depth and relatively low water volumes, shallow systems often experience rapid changes in water chemistry [63] and localized flushing during increased precipitation events [57,58,64,65,66]. Previous studies have confirmed the predominant influence of channel and flow characteristics, such as depth, over water quality on the success of some cyanobacterial species [67]. Additionally, some cyanobacterial species found in shallow environments may be more adapted than competitors because they are equipped to respond quickly to abrupt chemical changes and weather events—such as flash floods, high influxes of phosphate and nitrate, flash droughts, and warming—providing them with an advantage over competing species and allowing more frequent, dense CyanoHAB events [53,67,68,69].

The deep waterbodies seemed to be more stable [66] and had more consistent seasonal dissolved MC patterns with toxin concentrations peaking in summer and fall. Although the wetland sites had the highest measured total MC concentrations, the P-SL, R-JS, and P-TR sites had the most frequent detections, indicating more persistent toxin occurrence at these sites. Notably, the P-SL and P-TR sites are both shallow, manmade ponds primarily fed by storm runoff from adjacent golf courses and lack hydrologic connectivity. Similarly, the R-JS site is located where a river meets a reservoir, inherently diminishing water velocity and reducing flushing. In comparison, the wetland sites likely experienced frequent mixing and flow displacement events from seasonally variable inputs and runoff, making their bloom patterns more episodic and weather dependent [52,65,66,70]. In summary, HABs in shallow waterbodies—such as wetlands and ponds around north-central Tennessee—tend to develop and dissipate more frequently but often produce lower and less persistent dissolved toxin levels than those found in deep waterbodies [51,65,71,72]. These shallow systems are more sensitive to environmental variability and weather events, which can drive short-term fluctuations in bloom intensity and toxin release [51,52,58,63,65]. Previous studies have identified a relationship between residence times, flushing rates, and depth with CyanoHAB growth, but additional research would be needed to further explore the idea that shallow waterbodies lacking hydrologic connectivity or consistent flow produce higher toxin concentrations [16,36,73,74].

### 3.2. Drivers of Microcystin

The significant inverse correlation between MC concentrations and stream water levels (gage heights) is likely due to stormwater runoff processes caused during high precipitation events. Periods of heavy rain can mix and dilute cyanotoxins, scour periphyton from surfaces, increase turbulence, and flush cyanobacteria, hindering growth due to disturbance of water column stratification [56,58,68]. Lower water levels were correlated with less than average rainfall, typically during flash droughts, and caused increased residence times and reduced disturbance for sites with low hydrological connectivity. Seasonally reduced rainfall and diminished discharge encourage thermal stratification and warmer surface temperatures, conditions conducive for cyanobacteria growth and toxin production [71]. Tennessee often experiences seasonal flash floods and droughts, further demonstrating the potential utility of additional research aimed at fully characterizing the effects precipitation may have on cyanotoxin dynamics in freshwater systems.

PCA and logistic regression were applied to identify environmental parameters associated with the presence of cyanotoxins in the sampled north-central Tennessee locations. Specific conductance and pH showed a significant relationship with detectable MC occurrences; increases in both parameters correlated with a higher likelihood of detecting total MC (Table 8, Figure 7). It is important to note that the sample sizes were not large enough between site types to execute these analyses by waterbody type. The relationship between higher specific conductance and pH in freshwater ecosystems with increased cyanobacterial growth and cyanotoxin production agrees with findings from other studies [14,15,17,19], and specific conductance and pH are more sensitive to change in shallow environments [65,66,70], emphasizing the need to focus on specific ecosystem types to identify specific cyanobacterial species behavior, cyanobacterial growth and productivity, and cyanotoxin production and concentrations. These findings demonstrate that consistent sampling techniques, large sample sizes, and multivariate analysis could help increase understanding about the conditions that promote cyanobacterial toxin production and persistence across different waterbodies.

In conclusion, this study confirmed the widespread distribution and variable concentrations of cyanotoxins in north-central Tennessee and highlights the importance of ongoing monitoring, multivariate analysis, and site-specific context for interpreting cyanotoxin risks in freshwater systems. Complex environmental relationships, such as those involving water levels and water quality field data, require a robust, long-term sampling approach across a broad spatial scale to fully capture and understand patterns. Based on the findings of this study, we suggest that cyanotoxin occurrence in north-central Tennessee is shaped by waterbody type, environmental conditions, and hydrologic connectivity.

### 3.3. Future Research Directions

This study characterized the occurrence and distribution of multiple cyanotoxins across a variety of waterbody types in north-central Tennessee over a two-year period. This study was designed as an exploratory monitoring effort focused on cyanotoxin occurrence and distribution in north-central Tennessee, but significant relationships between cyanotoxins and environmental parameters were identified from the collected monitoring data. These findings add to the understanding of cyanotoxin distribution and occurrence in freshwater, variability in cyanotoxin production between different waterbody types, and the utility of applying multiple sampling approaches to such studies. Nutrient data were not collected for this study; therefore, the role of nitrogen and phosphorus in CyanoHAB formation were not explored. Future studies investigating CyanoHAB triggers could incorporate a nutrient monitoring component to better understand the specific dynamics of the resources which are needed for cyanotoxin production, particularly between nitrogen-fixing species and site characteristics. Future CyanoHAB monitoring studies could also consider higher spatial and temporal resolutions to further parse out these important relationships and differences between site types and increase sample sizes for statistical analyses, especially in larger waterbodies where one sampling site cannot adequately characterize the entire body. We acknowledge that sample sizes in the present study varied across sites due to differences in access, sampling windows, and available resources; however, these data still supported meaningful preliminary conclusions. Additionally, cyanobacterial species analysis could be considered to better understand the conditions favored for toxin production by specific cyanotoxin-producing species present during toxin detections.

## 4. Materials and Methods

### 4.1. Study Area and Site Descriptions

Monitoring locations comprised 18 sites in north-central Tennessee (Figure 1; Table 10). During site selection, locations near surface water utility intakes and high traffic recreational areas with safe access for SPATT sampler deployment and water collection were prioritized. Site types included lakes (2), rivers (6), wells (2), wetlands (6), and pond (2) sites (Figure 1; Table 10). Pond sites P-SL and P-TR are shallow, manmade ponds primarily fed by storm runoff from adjacent golf courses and lack hydrologic connectivity. During July 2022–November 2024, water quality field data, SPATT samplers, and discrete water samples were collected every two weeks (Table 10). For the 17 SPATT deployment sites, discrete water samples for total MC analysis and water quality field data were collected as SPATT samplers were deployed; the total numbers of water samples and SPATT samplers by site are provided in Table 10.

### 4.2. Field Sampling

Water quality data were collected during each site visit using a ProDSS Multi-Parameter Water Quality field meter (YSI Inc., Yellow Springs, OH, USA) and a Secchi tube. Calibration for the field meter was bracketed to the appropriate anticipated environmental field ranges on the morning of each field day as recommended by the manufacturer. Field readings included temperature (°C), pH, specific conductance (µS/cm), dissolved oxygen (mg/L), chlorophyll a (relative fluorescence units [RFUs]), phycocyanin (RFUs), and Secchi depth (cm). All cyanotoxin sample concentration values and water quality field data collected from this study are publicly available [47]. Total MC samples (discrete water samples) were collected in 50 mL high-density polyethylene (HDPE) plastic conical vials. The vials were rinsed three times with environmental field water before sample collection and were stored on ice in the field and immediately frozen (−20 °C) upon return to the laboratory. Water samples were frozen and thawed three times each to lyse the intracellular toxin from cyanobacterial cells into solution for total MC analysis [75]. These freeze–thaw steps rupture any present bacterial cells to release intracellular cyanotoxin into solution for analysis in addition to toxins already dissolved in solution; thus, measured toxins are referred to as total MC concentration.

Samples for time-integrated dissolved cyanotoxin concentrations were collected using SPATT samplers, which were passively deployed to capture potential dissolved cyanotoxin occurrences between site visits. SPATT samplers were deployed for approximately two weeks and exchanged upon retrieval. The samplers were constructed using HP20 resin (DIAION™, Mitsubishi Chemical Company, Tokyo, Japan) sealed with staples inside a folded milk sock (40–100 µm pore size; [76]). Constructed samplers were activated in 100% methanol and stored for 12–24 h [77]. After activation, SPATT samplers were rinsed with reverse osmosis (deionized) water to neutralize and effectively remove the methanol from the resin. The rinsed samplers were stored in deionized water and refrigerated (4 °C) until deployment. In the field, SPATT samplers were secured in the photic zone, near the water surface, using zip ties. The retrieved SPATT samplers were placed in labeled plastic bags and stored on ice in the field and frozen in the laboratory (−20 °C) until extraction.

### 4.3. Cyanotoxin Extraction and Analysis

To begin the extraction, SPATT samplers were removed from the freezer and disassembled, and resin contents were scraped into 250 mL glass flasks and mixed with 15 mL of 50% methanol and left in a laminar flow fume hood overnight to extract. After allowing the extraction to sit overnight, the resin-methanol mixture was gently swirled and gravity filtered into 20 mL glass test tubes through Whatman conical 90 mm glass microfiber filters with pore sizes less than 1 µm (GF/B; Cytiva, Kent, UK). A small amount of methanol (<5 mL) was used to rinse any remaining resin beads from the Erlenmeyer flasks onto the filter and to rinse the resin captured by the filter [78]. Once filtered, the methanol extracts were either evaporated down in a fume hood on a heated manifold at 40 °C (samples collected prior to April 2024) or dried using a drying oven at 100 °C (samples collected April 2024 and later). The methanol extracts were evaporated until ≤0.5 mL of extract remained in the test tube. The dried samples were reconstituted using 5 mL of ultrapure water, vortexed for 20 s, and placed in a sonic bath for 2 min to agitate and effectively redissolve cyanotoxin potentially sorbed to the test tube walls. After filtration, the remaining extracted resin from the SPATT sampler was dried for 72 h to then collect a final dry resin weight for toxin concentration calculations (µg/gram of resin) [77].

The SPATT sampler extracts and the unfiltered, lysed water samples were analyzed for anatoxin-a (Catalog [Cat] #—520060), cylindrospermopsin (Cat. #—22011), MC and nodularin (Cat. #—520011), and saxitoxin (Cat #—52255B) concentrations using enzyme linked immunosorbent assay (ELISA) kits (Gold Standard Diagnostics, Horsham, PA, USA). In addition to the calibration standards provided with each kit, 10% of the samples loaded onto each ELISA plate were sample duplicates or laboratory blanks to provide additional quality assurance. The calibration standards for all ELISA plates were analyzed according to the manufacturer for the associated cyanotoxin kit to assure <10% coefficient of variation between sample replicates and R^2^ ≥ 0.999 for all ELISA plate runs. Sample absorbances were analyzed using a Multiskan FC Microplate Photometer (Thermo Fisher, Waltham, MA, USA) at 450 nm. Eurofins Abraxis Solver calculation spreadsheets were used to calculate sample toxin concentrations from absorbances based on calibration standards (Gold Standard Diagnostics, Horsham, PA, USA). For samples with concentrations above the reporting limit, dilutions were performed using ultrapure water to bring the sample down to the reporting range and reanalyzed. The final cyanotoxin concentrations were multiplied by a dilution factor (if applicable) and divided by the dry resin weight to obtain a final relative concentration in µg/L per gram of resin extracted. All project monitoring data are publicly available via the following citation [47].

### 4.4. Cyanotoxin Biosynthesis Genes

Twelve of the 18 monitoring sites were sampled in August 2023 for qPCR analysis at the USGS Ohio Water Microbiology Laboratory (OWML) following the methods of [79,80]. Samples were collected in 200 mL poly bottles and stored on ice until arrival at the Tennessee State University (TSU) laboratory for filtration before shipment to the OWML for analysis [79]. In the TSU laboratory, 50–100 mL of the collected raw water was filtered through a 0.45 µm eDNA polycarbonate filter, and the filtered volume of sample was recorded for gene abundance calculations after analysis. Filters were placed into cryo-vials and stored at −80 °C until overnight shipment on dry ice to the OWML for analysis. The qPCR analysis was used to quantify the general cyanobacteria 16S gene abundance assay (16S) [81], cyanotoxin genes associated with MC (*mcyE*) [82], anatoxin (*anaC*) [83], cylindrospermopsin (*cyrA*) [82], and saxitoxin (*sxtA*) [82] (Table 11). OWML DNA-based qPCR analysis methods quantify the abundance (i.e., number of gene copies) of cyanobacteria known to possess the ability to produce cyanotoxin. Therefore, their presence in eDNA samples does not indicate the presence of cyanotoxins and would require additional sampling methods to confirm cyanotoxin presence at the time of eDNA sample collection. All eDNA assays were run on an Applied Biosystems QuantStudio™ 3 Real-Time PCR System at the OWML (ThermoFisher Scientific, Waltham, MA, USA). The OWML sample concentration, extraction, and qPCR methods (including primer, probe information, and run conditions) are available in the following citation [80].

### 4.5. Data Analysis

Continuous streamgage water level (gage height) data are publicly available from the USGS and were used to identify high and low precipitation periods from the R-SB sample location (USGS station 03431091, Cumberland River at Omohundro Water Plant at Nashville, TN, USA) [84]. Gage height data are publicly available from [48]. The R-SB site is located on the Cumberland River, which also has a significant hydrologic connection to many of the other monitoring locations in this study. The river water level is partially controlled by two reservoirs immediately upgradient of the R-SB site. However, heavy rains or long dry spells exert an influence on the streamgage water level and provide insight into major weather patterns.

All data organization and statistical analyses on cyanotoxin concentrations and water quality data were performed using RStudio [85] version 2024.12.1 + 563 “Kousa Dogwood” Release for Windows 11OS [86]. Statistics performed in Rstudio included descriptive statistics for site characterization and variability, Kendall’s Tau correlation test to analyze for correlations of environmental parameters with MC concentrations, a principal component analysis (PCA), logistic regression, and the general variance test applied to the PCA results. The PCA was conducted using the factoextra R package version 1.0.7 [87] to condense water quality data from the various site types and chemical conditions into components representing the main sources of variability. PCA data were log(x + 1) transformed and normalized to mean and standard deviation, and a resemblance matrix was calculated based on Euclidean distance. Correlation matrices were generated using version 0.95 of the corrplot package in R [88].

A logistic regression analysis was also conducted using MC detections and the PCA scores from the water quality data to determine if there were significant environmental variables (temperature, dissolved oxygen, specific conductivity, chlorophyll a fluorescence, phycocyanin fluorescence, pH, and Secchi depth) that predicted the probability of MC presence (all instances of detectable concentrations) or absence (all instances of no detected concentrations). Plots were produced using the ggplot2 package version 3.5.2 [89] in Rstudio; site maps and eDNA data spatial projections were symbolized and exported from ArcGIS Pro 3.4.0 (Esri, Redlands, CA, USA).

## Figures and Tables

**Table 1:** Summary of dissolved microcystin (MC) concentrations from SPATT (Solid Phase Adsorption Toxin Tracking) samplers and total MC concentrations from discrete water samples collected during October 2022–November 2024. The period sampled dates are stated in month, day, year format (i.e. MM/DD/YYYY). The number of observations (*n*) and percentage of samples with detectable MC (% Pos.) are provided from all sites. Maximum (Max.), median (Med.), and standard error (SE) values are provided for sites with measurements above the lower MC reporting limit (0.150 µg/L). “<RL” indicates values were below the lower reporting limit (0.150 µg/L) and “>RL” indicates that MC concentrations exceeded the upper reporting limit (5.0 µg/L for total MC, 25.0 µg/L for dissolved MC). “-” indicates that samples were not analyzed for that toxin at that site. The associated toxin concentration data used to generate these values is publicly available from [47].

		Period Sampled		Dissolved MC (µg/g)					Total MC (µg/L)				
Environment	Site	Start	End	*n*	% Pos.	Max.	Med.	SE	*n*	% Pos.	Max.	Med.	SE
Lake	L-JPP	10/31/2022	07/30/2024	15	100	69.4	21.1	5.4	19	10.5	0.16	<RL	0.0
Lake	L-OH	07/10/2024	11/27/2024	10	100	12.8	2.35	1.1	10	10.0	0.48	<RL	0.0
Pond	P-SL	06/22/2023	11/27/2024	23	100	15.5	2.60	0.9	30	20.0	0.84	<RL	0.0
Pond	P-TR	10/28/2022	11/27/2024	41	87.8	38.4	1.10	1.0	41	14.6	0.27	<RL	0.0
River	R-GG	10/14/2022	11/27/2024	30	100	21.5	3.45	0.9	35	5.70	0.17	<RL	0.0
River	R-JS	11/02/2022	12/06/2023	12	100	10.7	3.10	0.8	16	18.8	0.32	<RL	0.0
River	R-SB	11/02/2022	11/27/2024	27	96.3	55.8	4.30	2.2	34	0.0	<RL	<RL	0.0
River	R-WH	09/30/2022	12/06/2023	14	100	12.7	1.45	1.0	18	0.0	<RL	<RL	0.0
Well	G-GU	11/03/2022	12/06/2023	15	100	21.1	3.40	1.5	18	0.0	<RL	<RL	0.0
Well	G-GW	10/14/2022	04/18/2024	21	61.9	1.30	0.33	0.1	22	0.0	<RL	<RL	0.0
Wetland	W-BP	09/23/2022	11/14/2024	37	75.7	20.1	0.67	0.7	42	11.9	>RL	<RL	0.1
Wetland	W-BWL	09/23/2022	11/27/2024	40	97.5	21.1	2.15	0.7	42	2.40	0.16	<RL	0.0
Wetland	W-EWL	09/23/2022	04/18/2024	30	90.0	9.50	1.40	0.4	33	12.1	0.28	<RL	0.0
Wetland	W-HP	06/22/2023	04/18/2024	13	76.9	2.40	0.63	0.2	18	0.0	<RL	<RL	0.0
Wetland	W-MWL	09/23/2022	11/27/2024	37	86.5	23.1	1.00	1.0	43	11.6	2.00	<RL	0.1
Wetland	W-TDA	08/24/2023	04/18/2024	0	-	-	-	-	11	0.0	<RL	<RL	0.0
Wetland	W-TDI	10/04/2023	04/18/2024	11	90.9	5.40	0.69	0.5	13	0.0	<RL	<RL	0.0
Wetland	W-UWL	09/23/2022	11/27/2024	44	77.3	10.4	0.69	0.3	44	11.4	>RL	<RL	0.1

**Table 2:** Summary of dissolved toxin concentrations (µg/g) of anatoxin, cylindrospermopsin, and saxitoxin from SPATT samplers collected during August 2023–November 2024. The period sampled dates are stated in month, day, year format (i.e. MM/DD/YYYY). The number of observations (*n*), percentage of SPATT samplers with detectable toxin (% Pos.), maximum (Max.), median (Med.), and standard error (SE) are provided for each toxin. “<RL” indicates values were below the respective lower reporting limit (0.150 µg/L for anatoxin, 0.050 µg/L for cylindrospermopsin, and 0.020 µg/L for saxitoxin) and “>RL” indicates concentrations were detected above the upper reporting limit (25.0 µg/L for anatoxin, 10.0 µg/L for cylindrospermopsin, and 2.00 µg/L for saxitoxin). “-” indicates that samples were not analyzed for that toxin at that site. The associated toxin concentration data used to generate these values are publicly available from [47].

	Period Sampled		Anatoxin (µg/g)					Cylindrospermopsin (µg/g)					Saxitoxin (µg/g)				
Site	Start	End	*n*	% Pos.	Max.	Med.	SE	*n*	% Pos.	Max.	Med.	SE	*n*	% Pos.	Max.	Med.	SE
L-JPP	09/05/2023	04/04/2024	6	100	>RL	11.5	1.5	0	-	-	-	-	2	0.0	<RL	<RL	0.0
L-OH	10/17/2024	11/27/2024	4	100	0.78	0.60	0.1	0	-	-	-	-	4	25.0	0.49	<RL	0.1
P-SL	09/13/2023	11/27/2024	12	83.3	10.9	2.35	0.9	3	100	1.30	1.30	0.0	12	25.0	0.16	<RL	0.0
P-TR	08/24/2023	11/27/2024	11	90.9	>RL	1.48	2.4	2	100	1.50	1.35	0.2	14	28.6	0.65	<RL	0.0
R-GG	08/24/2023	11/27/2024	10	100	>RL	5.00	3.4	0	-	-	-	-	12	0.0	<RL	<RL	0.0
R-JS	08/30/2023	11/08/2023	5	100	>RL	8.50	3.1	0	-	-	-	-	1	0.0	<RL	<RL	-
R-SB	09/13/2023	11/27/2024	11	100	>RL	1.00	2.2	1	100	1.70	1.70	-	10	20.0	0.26	<RL	0.0
R-WH	09/30/2022	12/06/2023	7	100	>RL	8.00	2.9	0	-	-	-	-	2	0.0	<RL	<RL	0.0
G-GU	08/30/2023	11/22/2023	4	100	>RL	12.0	3.3	0	-	-	-	-	1	0.0	<RL	<RL	-
G-GW	08/24/2023	03/20/2024	8	37.5	5.00	1.00	0.6	1	0.0	<RL	<RL	-	6	33.3	0.20	<RL	0.0
W-BP	08/24/2023	10/17/2024	10	80.0	>RL	3.40	2.4	2	100	1.40	1.16	0.2	8	25.0	0.45	<RL	0.1
W-BWL	08/24/2023	11/27/2024	12	91.7	20.1	2.50	1.7	3	100	4.60	3.80	0.6	13	38.5	0.45	<RL	0.1
W-EWL	08/24/2023	04/04/2024	7	85.7	>RL	3.40	3.7	3	100	2.20	1.60	0.3	6	66.7	0.25	<RL	0.0
W-HP	08/30/2023	04/04/2024	7	28.6	5.10	2.03	0.9	3	100	1.70	0.98	0.3	6	33.3	0.22	0.13	0.0
W-MWL	08/24/2023	11/27/2024	12	91.7	17.1	2.85	1.5	3	100	2.90	1.10	0.6	11	45.5	0.37	<RL	0.0
W-TDA	-	-	0	-	-	-	-	0	-	-	-	-	0	-	-	-	-
W-TDI	10/18/2023	03/07/2024	0	-	-	-	-	0	-	-	-	-	9	33.3	0.43	<RL	0.1
W-UWL	08/30/2023	11/22/2023	15	100	>RL	4.00	2.0	2	100	1.00	0.92	0.0	14	14.3	0.33	<RL	0.0

**Table 3:** Summary of dissolved MC concentrations from SPATT samplers and total MC concentrations from discrete water samples collected during October 2022–November 2024. The number of observations per month (*n*), percentages of samples with detectable MC (% Pos.), maximum (Max.), median (Med.), and standard error (SE) are provided. “<RL” indicates values were below the lower reporting limit (0.150 µg/L) and “>RL” indicates that MCs were detected but above the upper reporting limit (5.0 µg/L). The associated toxin concentration data used to generate these values are publicly available from [47]. Water levels (gage heights) from USGS streamgage 03431091 were also pooled to calculate monthly maximum (Max.) and mean streamgage water level in meters (m) as an indicator of precipitation. Gage height data are publicly available from [48].

	Dissolved MC (µg/g)					Total MC (µg/L)					Water Level (m)		
Month	*n*	% Pos.	Max.	Med.	SE	*n*	% Pos.	Max.	Med.	SE	Max.	Mean	SE
January	17	88.2	4.30	0.81	0.3	25	0.0	<RL	<RL	0.0	8.3	6.6	0.02
February	31	71.0	8.80	0.37	0.4	26	0.0	<RL	<RL	0.0	9.0	7.2	0.04
March	33	72.7	3.00	0.51	0.1	38	7.89	>RL	<RL	0.2	9.1	7.1	0.03
April	33	78.8	13.5	0.81	0.4	38	0.0	<RL	<RL	0.0	7.7	6.4	0.02
May	12	75.0	1.20	0.26	0.1	12	0.0	<RL	<RL	0.0	12.3	7.3	0.07
June	25	88.0	16.1	1.20	0.7	38	13.2	2.00	<RL	0.1	8.1	6.4	0.03
July	37	94.6	35.1	3.60	1.3	52	7.69	0.84	<RL	0.0	7.1	6.2	0.01
August	49	98.0	66.2	2.90	1.5	55	10.9	0.48	<RL	0.0	6.8	6.2	0.01
September	48	97.9	69.4	1.95	1.5	50	12.0	1.30	<RL	0.0	7.0	6.1	0.01
October	55	87.3	27.9	2.30	0.9	55	1.82	0.18	<RL	0.0	6.4	6.0	0.01
November	66	98.5	55.8	2.20	1.2	84	16.7	0.32	<RL	0.0	6.3	6.0	0.01
December	14	92.9	15.5	0.62	1.2	16	6.25	0.15	<RL	0.0	8.8	6.6	0.03

**Table 4:** Summary of water quality field data collected from 18 sites during August 2022–November 2024. The period sampled, number of observations (*n*), median (Med.), and standard error (SE) values are provided. The period sampled dates are stated in month, day, year format (i.e. MM/DD/YYYY). Provided water quality data include temperature (Temp.), dissolved oxygen concentration (DO), specific conductance (SC), chlorophyll a (Chl-a), phycocyanin (PC), pH, and Secchi depth (Secchi, “>RL” ≥ 120 cm). Secchi depth data were not collected at groundwater sites (G-GU and G-GW) as indicated by “-”. The associated water quality field data used to generate these values are publicly available from [47].

		Period Sampled			Temp. (°C)		DO (mg/L)		SC (µS/cm)		Chl-a (RFUs)		PC (RFUs)		pH		Secchi (cm)	
Environment	Site	Start	End	*n*	Med.	SE	Med.	SE	Med.	SE	Med.	SE	Med.	SE	Med.	SE	Med.	SE
Lake	L-JPP	10/31/2022	7/30/2024	20	24.4	1.6	8.80	0.5	279	7.0	1.20	0.3	0.30	0.1	8.2	0.1	99	4.1
Lake	L-OH	7/10/2024	11/27/2024	12	25.4	1.7	9.40	0.8	200	2.5	2.50	0.5	0.90	0.1	8.3	0.2	66	4.5
Pond	P-SL	6/22/2023	11/27/2024	35	20.4	1.5	10.4	0.8	392	7.0	8.10	1.6	2.30	0.4	8.4	0.1	32	2.8
Pond	P-TR	10/28/2022	11/27/2024	47	19.2	1.2	9.50	0.4	322	18.3	3.70	2.2	1.10	0.4	8.2	0.1	37	3.1
River	R-GG	10/14/2022	11/27/2024	39	18.7	1.1	8.70	0.3	216	3.2	2.00	0.6	0.50	0.1	7.9	0.0	62	4.6
River	R-JS	11/2/2022	12/6/2023	18	24.9	1.5	9.90	0.5	367	21.4	2.70	0.3	0.80	0.1	8.1	0.1	40	4.1
River	R-SB	11/2/2022	11/27/2024	41	19.4	1.1	8.65	0.4	209	3.2	1.70	0.4	0.40	0.0	7.9	0.0	63	3.8
River	R-WH	9/30/2022	12/6/2023	20	19.7	1.2	7.60	0.6	408	9.4	1.80	0.6	0.20	0.1	7.8	0.1	65	7.9
Well	G-GU	11/3/2022	12/6/2023	21	17.8	0.4	5.55	0.5	593	38.9	0.10	0.4	0.00	0.0	7.0	0.1	-	-
Well	G-GW	10/14/2022	4/18/2024	25	17.3	0.5	2.40	0.2	575	18.1	0.00	0.1	0.00	0.0	7.4	0.0	-	-
Wetland	W-BP	8/25/2022	11/27/2024	51	18.1	1.2	11.2	0.7	477	20.9	5.10	1.7	1.30	0.4	8.1	0.1	58	7.3
Wetland	W-BWL	8/25/2022	11/27/2024	51	17.8	1.0	7.40	0.6	567	15.2	0.50	0.3	0.10	0.0	7.7	0.0	>RL	5.8
Wetland	W-EWL	8/25/2022	4/18/2024	38	17.9	1.1	8.90	0.6	605	12.8	2.15	0.6	0.70	0.2	7.6	0.1	100	6.5
Wetland	W-HP	6/22/2023	4/18/2024	24	15.6	2.1	6.60	1.1	133	38.7	7.30	4.6	2.35	5.1	6.8	0.1	11	3.7
Wetland	W-MWL	8/25/2022	11/27/2024	52	17.6	1.1	13.7	0.8	541	15.7	3.25	3.0	0.75	1.5	8.2	0.1	89	6.0
Wetland	W-TDA	4/19/2023	4/18/2024	28	20.2	1.7	8.55	0.7	534	27.9	1.25	4.1	0.50	0.3	7.7	0.0	>RL	9.2
Wetland	W-TDI	10/4/2023	4/18/2024	15	13.3	1.2	12.2	0.4	509	14.8	1.70	0.7	0.40	0.2	8.1	0.1	>RL	8.9
Wetland	W-UWL	8/25/2022	11/27/2024	51	17.2	0.7	6.35	0.6	630	10.6	2.40	1.6	0.80	0.8	7.3	0.0	68	6.2

**Table 5:** General variance test results from principal component analysis (PCA) results between site depth groups. Gen. Var. = variance using PC1 and PC2; Top-k = variance using top-k PCs that explain approximately 85% of the variance of the data. Greater values represent higher variance.

Depth Group	Gen. Var.	Top-k
Shallow	1.91	1.6028
Deep	0.28	0.0383
Well	0.01	0.0001

**Table 6:** Summary of water quality field data collected during August 2022–November 2024, pooled by month. The period sampled, number of observations (*n*), maximum (Max.; reported for temperature only), median (Med.), and standard error (SE) values are provided. The period sampled dates are stated in month, day, year format (i.e. MM/DD/YYYY). Provided water quality data include temperature (Temp.), dissolved oxygen (DO), specific conductance (SC), chlorophyll a (Chl-a), phycocyanin (PC), pH, and Secchi depth (Secchi, “>RL” = > 120 cm). The associated water quality field data used to generate these values are publicly available from [47]. Water levels (gage heights) from USGS streamgage 03431091 were also pooled to calculate monthly maximum (Max.) and mean streamgage water level in meters (m) as an indicator of precipitation. Gage height data are publicly available from [48].

	Period Sampled			Temp. (°C)			DO (mg/L)		SC (µS/cm)		Chl-a (RFUs)		PC (RFUs)		pH		Secchi (cm)		Water Level (m)		
Month	Start	End	*n*	Max.	Med.	SE	Med.	SE	Med.	SE	Med.	SE	Med.	SE	Med.	SE	Med.	SE	Max.	Mean	SE
January	1/10/2023	1/24/2024	32	13.7	8.00	0.6	12.2	0.5	558	33.6	3.5	2.7	0.8	1.4	7.8	0.1	74	6.1	8.25	6.6	0.02
February	2/7/2023	2/22/2024	37	18.3	13.0	0.4	11.7	0.5	630	30.9	2.3	1.1	0.9	0.3	7.9	0.1	111	6.6	8.98	7.2	0.04
March	3/7/2023	3/20/2024	38	20.0	16.9	0.3	11.4	0.8	530	31.7	1.8	2.5	0.6	0.4	8.1	0.1	>RL	6.2	9.09	7.1	0.03
April	4/4/2023	4/18/2024	39	26.0	19.3	0.6	9.10	0.6	523	22.5	1.7	2.9	0.5	2.5	7.8	0.1	90	7.1	7.69	6.4	0.02
May	5/3/2023	5/18/2023	14	27.4	20.4	1.0	10.5	1.0	488	23.5	1.0	0.2	0.4	0.4	8.2	0.2	63	14.0	12.3	7.3	0.07
June	6/1/2023	6/17/2024	41	34.2	25.0	0.8	7.90	0.6	398	20.6	1.6	1.4	0.5	0.4	7.8	0.1	69	6.6	8.12	6.4	0.03
July	7/6/2023	7/31/2024	56	33.6	26.9	0.6	7.00	0.5	349	23.7	1.7	2.5	0.6	1.8	7.7	0.1	61	6.4	7.07	6.2	0.01
August	8/25/2022	8/29/2024	65	32.6	26.2	0.4	6.30	0.5	362	20.9	2.8	1.7	1.0	0.2	7.8	0.1	44	3.9	6.76	6.2	0.01
September	9/23/2022	9/19/2024	66	30.2	24.6	0.5	6.80	0.5	396	21.8	2.9	2.0	0.8	0.8	7.6	0.1	55	5.5	6.96	6.1	0.01
October	10/14/2022	10/31/2024	76	26.3	18.7	0.4	7.90	0.5	450	22.2	3.1	0.9	0.6	0.3	7.9	0.1	45	5.2	6.45	6.0	0.01
November	11/2/2022	11/27/2024	95	19.3	14.5	0.4	8.90	0.4	436	19.8	2.0	0.5	0.4	0.1	7.8	0.1	65	4.3	6.31	6.0	0.01
December	12/6/2023	12/20/2023	29	19.7	8.70	0.7	10.9	0.6	548	24.8	3.1	0.9	0.7	0.2	7.9	0.1	>RL	7.3	8.77	6.6	0.03

**Table 7:** Abundance (copies/100 mL) of cyanobacteria (16S) and microcystin (*mcyE*), anatoxin (*anaC*), cylindrospermopsin (*cyrA*), and saxitoxin (*sxtA*) synthetase genes in water samples collected in August 2023 from 12 sites. <QL indicates that the sample was below the quantification limit (1100 copies/100 mL for *mcyE*, 60 copies/100 mL for *anaC*, 120 copies/100 mL for *cyrA*, and 300 copies/100 mL for *sxtA*). The date sampled dates are stated in month, day, year format (i.e. MM/DD/YYYY). “E” indicates the value was estimated due to low abundance values—refer to Methods section and References for details. The associated gene abundance data used to generate this table are publicly available from [47].

Gene Abundance (Copies 100 mL^−1^)											
Site	Date Sampled	Volume Filtered (mL)	16S	*mcyE*		*anaC*		*cyrA*		*sxtA*	
L-JPP	08/21/2023	100	3.90 × 10^8^	1500	E	<QL		<QL		<QL	
P-SL	08/17/2023	50	5.00 × 10^8^	330,000		5400		<QL		18,000	
P-TR	08/24/2023	100	1.10 × 10^9^	69,000		<QL		<QL		940	E
R-GG	08/24/2023	100	5.10 × 10^8^	210,000		5800		<QL		33,000	
R-JS	08/17/2023	50	2.10 × 10^9^	14,000	E	2500		<QL		4,700,000	
R-SB	08/17/2023	100	2.00 × 10^8^	480,000		6400		1500	E	95,000	
R-WH	08/17/2023	100	6.10 × 10^7^	6900		520	E	<QL		30,000	
G-GU	08/17/2023	100	5.50 × 10^6^	2100	E	510	E	980	E	<QL	
W-BP	08/24/2023	50	1.40 × 10^8^	4400	E	3700		<QL		16,000	
W-BWL	08/24/2023	100	2.40 × 10^7^	<QL		440	E	<QL		600	E
W-MWL	08/24/2023	50	2.20 × 10^9^	<QL		1300	E	1200	E	13,000	
W-UWL	08/24/2023	100	1.80 × 10^6^	<QL		8500		600	E	<QL	

**Table 8:** Dominant (correlation > 0.5) eigenvector values from principal component analysis (PCA). PC = principal component.

PC	Eigenvector Variable(s)	% Variation	Cumulative % Variation
1	Secchi depth	31.6	31.6
2	Chlorophyll a	22.5	54.1
3	pH	18.8	72.9
4	Temperature, conductivity	15.8	88.6

**Table 9:** Odds ratios determined from a logistic regression model using the presence and absence of total MC as the response variable. Odds ratios values that are greater than 1 indicate that MC is more likely to occur as the predictor variable increases. Bold lettering indicates a significant result (*p* < 0.05).

Predictor Variable	Odds Ratio	*p*
Temperature	1.025	0.276
Dissolved oxygen	0.963	0.431
**Specific conductance**	**1.003**	**0.004**
Chlorophyll a fluorescence	1.017	0.509
Phycocyanin fluorescence	1.025	0.798
**pH**	**2.260**	**0.025**
Secchi depth	0.995	0.208

**Table 10:** Table of 18 monitoring sites with site types, site IDs, depth groups (based on wadable vs. non-wadable) site names, U.S. Geological Survey (USGS) station numbers [48], and total sample counts for discrete water samples and SPATT samplers collected.

Site Type	Site ID	Depth Group	Site Name	USGS Station Number	Water	SPATT
Lake	L-JPP	Deep	J Percy Priest Dam	03430045	19	15
Lake	L-OH	Deep	Old Hickory Rockland Rec. Area	361759086384701	10	10
Pond	P-SL	Deep	Shelby Bottoms Sevier Lake	361012086434901	30	23
Pond	P-TR	Deep	Ted Rhodes Upstream Pond	361116086493001	41	41
River	R-GG	Deep	Ted Rhodes USGS Gage	03431514	35	30
River	R-JS	Deep	Jefferson Springs Rec. Area	03429010	16	12
River	R-SB	Deep	Shelby Bottoms USGS Gage	03431091	34	27
River	R-WH	Deep	Walter Hill Rec. Area	03427727	18	14
Well	G-GU	Well	Gladeville Utility District	360346086250101	18	15
Well	G-GW	Well	TSU Research Well	361036086493901	22	21
Wetland	W-BP	Shallow	Bull Pond TSU Wetland	0343151501	42	37
Wetland	W-BWL	Shallow	Below TSU Wetland	0343151504	42	40
Wetland	W-EWL	Shallow	Experimental TSU Wetland	0343151500	33	30
Wetland	W-HP	Shallow	Shelby Bottoms Hidden Pond	361026086493601	18	13
Wetland	W-MWL	Shallow	Mid TSU Wetland	0343151502	43	37
Wetland	W-TDA	Shallow	TSU Wetland Dam	0343151503	11	0
Wetland	W-TDI	Shallow	TSU Ditch near Wetland	361021086493701	13	11
Wetland	W-UWL	Shallow	Upper TSU Wetland	361028086492601	44	44

**Table 11:** qPCR analysis standard curve characteristics (dynamic range of quantification, amplification efficiency percent, R^2^ value), limits of detection (LoD), and limit of quantification (LoQ), for cyanobacteria assays. Cyanobacteria quantification units are copies/qPCR reaction (i.e., dynamic range, LoD, LoQ).

Gene Assay	Dynamic Range	Amplification Efficiency	R^2^	LoD	LoQ
16S	50.1–5.01 × 10^7^	95%	0.997	114	180
*mcyE*	16.1–1.61 × 10^7^	97%	0.998	27	270
*anaC*	50.0–5.00 × 10^7^	91%	0.999	3	54
*cyrA*	30.7–3.07 × 10^7^	96%	0.998	3	27
*sxtA*	65.6–6.56 × 10^7^	93%	0.997	15	120

## Data Availability

The original data presented in the study are openly available in the U.S. Geological Survey Science Base Catalog at https://doi.org/10.5066/P13Q2TD5 (accessed 22 May 2025).

## Abbreviations

The following abbreviations are used in this manuscript:

*anaC*	Anatoxin synthetase gene
Chl-a	Chlorophyll A
Cm	Centimeters
*cyrA*	Cylindrospermopsin synthetase gene
CyanoHABs	Cyanobacterial harmful algal blooms
DO	Dissolved oxygen
E	Estimated
HAB	Harmful algal bloom
LoD	Limit of detection
LoQ	Limit of quantification
Max.	Maximum value
MC	Microcystin
M	Meters
Med.	Median value
*mcyE*	Microcystin synthetase gene
mL	Milliliters
ND	No data
PC	Phycocyanin
PC	Principal component
PCA	Principal component analysis
QL	Quantification limit
qPCR	Quantitative polymerase chain reaction
RFUs	Relative fluorescent units
RL	Reporting limit
Rrna	Ribosomal RNA
*sxtA*	Saxitoxin synthetase gene
SC	Specific conductivity
SE	Standard error
SPATT	Solid Phase Adsorption Toxin Tracking
TDEC	Tennessee Department of Environment and Conservation
Temp	Temperature
TN	Tennessee
TSU	Tennessee State University
USGS	U.S. Geological Survey
µg/g	Micrograms per gram
µg/L	Micrograms per liter
µS/cm	Microsiemens per centimeter
°C	Degrees Celsius
